# Relationship between Stereoscopic Vision, Visual Perception, and Microstructure Changes of Corpus Callosum and Occipital White Matter in the 4-Year-Old Very Low Birth Weight Children

**DOI:** 10.1155/2015/842143

**Published:** 2015-09-16

**Authors:** Przemko Kwinta, Izabela Herman-Sucharska, Anna Leśniak, Małgorzata Klimek, Paulina Karcz, Wojciech Durlak, Magdalena Nitecka, Grażyna Dutkowska, Anna Kubatko-Zielińska, Bożena Romanowska-Dixon, Jacek Józef Pietrzyk

**Affiliations:** ^1^Department of Pediatrics, Jagiellonian University, Wielicka 265, 30-663 Cracow, Poland; ^2^Department of Electroradiology, Jagiellonian University, Michalowskiego 12, 31-126 Cracow, Poland; ^3^Department of Opthalmology and Ocular Oncology, Jagiellonian University, Kopernika 36, 31-501 Cracow, Poland; ^4^Department of Applied Psychology and Human Development, Jagiellonian University, Wielicka 265, 30-663 Cracow, Poland

## Abstract

*Aim.* To assess the relationship between stereoscopic vision, visual perception, and microstructure of the corpus callosum (CC) and occipital white matter, 61 children born with a mean birth weight of 1024 g (SD 270 g) were subjected to detailed ophthalmologic evaluation, Developmental Test of Visual Perception (DTVP-3), and diffusion tensor imaging (DTI) at the age of 4.* Results*. Abnormal stereoscopic vision was detected in 16 children. Children with abnormal stereoscopic vision had smaller CC (CC length: 53 ± 6 mm versus 61 ± 4 mm; *p* < 0.01; estimated CC area: 314 ± 106 mm^2^ versus 446 ± 79 mm^2^; *p* < 0.01) and lower fractional anisotropy (FA) values in CC (FA value of rostrum/genu: 0.7 ± 0.09 versus 0.79 ± 0.07; *p* < 0.01; FA value of CC body: 0.74 ± 0.13 versus 0.82 ± 0.09; *p* = 0.03). We found a significant correlation between DTVP-3 scores, CC size, and FA values in rostrum and body. This correlation was unrelated to retinopathy of prematurity.* Conclusions*. Visual perceptive dysfunction in ex-preterm children without major sequelae of prematurity depends on more subtle changes in the brain microstructure, including CC. Role of interhemispheric connections in visual perception might be more complex than previously anticipated.

## 1. Introduction

Preterm birth and very low birth weight (VLBW) increase the risk of visual sensory and perceptive dysfunctions [1]. Retinopathy of prematurity (ROP) contributes to significant level of visual impairment in VLBW children [2]. However, ROP does not explain the full range of visual impairments observed in these children. Visual perceptual disturbances have been reported in preterm children without ROP [3] as well as in late preterm children without brain injury [4]. They were related to macroscopic brain abnormalities including periventricular leukomalacia (PVL), occipital damage caused by hypoxia, or hydrocephalus. Van den Hout et al. [5] reported strong relationship between visual perceptual deficits and PVL, but not IVH alone in a group of preterm children assessed at the age of five. Interestingly, children with intact splenium of the corpus callosum (CC) had a better visual prognosis than those with CC injury [5]. The importance of central gray matter damage, thalamic atrophy, and cerebral white matter (WM) injury has also been reported in the context of abnormal neurodevelopment [6, 7]. The link between tissue architecture of the optic radiation and visual function in preterm neonates has been previously reported [8]. All these abnormalities have been implicated in visual perception and Visual-Motor Integration development, the two qualities that are crucial for proper intellectual development of a child and have been shown to be impaired in preterm born children [9]. Deficits in these skills could contribute to reported IQ differences between preterm and term children [10].

Magnetic resonance imaging has contributed to better understanding of the relationships between macroscopic structural abnormalities, like PVL and visual impairment in prematurity survivors. Conventional MR imaging has shown wide range of differences in preterm brain, including WM and gray matter volumes, CC abnormalities, and lateral ventricles enlargement [11]. Correlation between visual perception deficits and structural abnormalities diagnosed with conventional MR has been reported in a group of prematurity survivors with parenchymal brain hemorrhage and PVL [12].

However, not all developmental pathologies could be correlated with structural abnormalities in conventional MRI. Diffusion tensor imaging (DTI) provides higher sensitivity and accuracy of WM imaging. The primary principle of DTI is based on measurement of water diffusion in the tissue. Water diffusion in cerebral WM is determined by tissue architecture. Therefore DTI is highly sensitive to WM microstructural changes. Fractional anisotropy (FA) is a value derived from axial (along the axons) and radial (perpendicular) diffusivity. It represents values between 0 and 1 and is considered to correlate with WM maturity [13, 14].

The role of CC in visual integration has been previously reported in studies describing structural organization of V1 visual projections within the splenium both in humans [15–17] and in animal models [18, 19]. Structural abnormalities of visual splenial fibers have been implicated in pathophysiology of higher brain functions involving visual integration, such as alexia [20]. Recently published data suggests that less efficient visual processing might be related to reduced WM integrity in other parts of the CC, for example, body [21]. Thus, it seems reasonable to verify the role of callosal WM integrity in visual perception of VLBW children.

The primary aim of the study was to assess the relationship between brain microstructure (including CC and occipital WM), stereoscopic vision, and visual perception in a cohort of VLBW children at the age of 4 years. The results of the following methods have been compared: DTI, detailed ophthalmologic evaluation, and Developmental Test of Visual Perception (DTVP).

## 2. Material

A prospective study was conducted in the Follow-Up Pediatric Department of the Polish-American Children's Hospital, Cracow, Poland, between February 1, 2013, and January 31, 2015.

From 1 October 2008 to 31 October 2010, one hundred and four newborns with birth weight from 500 to 1500 g were discharged home form Neonatal Intensive Care Unit (NICU) of the Polish-American Children's Hospital. All of those alive at the age of 4–4.5 years were invited to participate in the follow-up study (*n* = 101). Neonatal data used for the study was recorded during NICU's stay daily in a prospective manner and stored in the computer databases. For the purpose of the study, the following data was extracted from original databases: gender, birth weight, gestational age, intrauterine growth, Apgar score, incidence of preeclampsia, premature rapture of the membranes, chorioamnionitis, presence of respiratory distress syndrome (RDS), need for mechanical ventilation, surfactant administration, use of ibuprofen for patent ductus arteriosus (PDA), PDA ligation, early and late onset sepsis episodes, prevalence and the grade of intraventricular hemorrhage (IVH), PVL, bronchopulmonary dysplasia (BPD), defined as at least 28 days of oxygen therapy and also defined as oxygen therapy at the 36-week postmenstrual age (PMA), weight gain during NICU stay, and length of hospitalization.

The Ethical Committee for Clinical Investigations of Collegium Medicum, Jagiellonian University, approved the study protocol.

## 3. Methods

After signing the informed consent by the parents, detailed ophthalmologic evaluation, DTVP, and DTI were performed in all children.

### 3.1. Ophthalmologic Evaluation

In order to assess stereoscopic vision, we used 3 stereopsis tests: the Lang test, TNO, and the Titmus test [22]. The Lang test is especially useful in assessing stereopsis in very young children and babies. It uses two images, which are separately seen by each eye when focused through a series of fine cylindrical lens elements. Displacement of the dots creates disparity. The patient is asked to name or point to a simple shape on the card. The examiner can also observe the child's eye movements from picture to picture on the card. The degree of disparity is quite gross, ranging from 1200 to 600 seconds of arc. This test does not require special spectacles. The TNO test is a random dot test consisting of seven plates, which are viewed with red-green spectacles. Each plate contains various shapes created by random dots in complementary colors. Some shapes are visible even without red-green spectacles, while others are hidden and only apparent to an individual with stereopsis wearing red-green spectacles. The degree of disparity ranges from 480 to 15 seconds of arc.

The Titmus test consists of a three-dimensional Polaroid vectograph consisting of two plates in the form of a booklet, which is viewed through Polaroid spectacles. On the right, there is a large housefly, and on the left there are series of circles and cartoons. The fly is a test of gross stereopsis (3000 seconds of arc). Children are often tested by asking them to hold one of the wings of the fly, which they will do above the plate if it is seen stereoscopically. The other vectograms of the test provide finer tests for stereoscopic acuity. In the circle test, the degree of disparity ranges from 800 to 40 seconds of arc and in the animal test from 400 to 100 seconds of arc.

Two experienced ophthalmologists performed all three tests separately in all children. The diagnosis was made after careful discussion between both examiners.

All patients were divided into two groups. The first group was composed of children with stereopsis (at least one of the performed tests was positive). Children without stereopsis were classified into the second group.

### 3.2. DTVP

Visual perceptive abilities were examined using the DTVP-3, most recent revised version of the classic Marianne Frostig DTVP. All children were examined using five subtests. In the Eye-Hand Coordination Test, they were asked to draw straight or curved lines according to given boundaries. The Copying Test aimed at drawing simple figures shown to the patient in a piece of paper; subsequent figures were arranged in the order of increasing complexity. In the Figure-Ground Test, children were asked to find as many simple figures as possible hidden in a complex background. During the Visual Closure test, children were shown a stimulus figure and asked to choose a corresponding one from a series of incompletely drawn figures. Finally in the Form Constancy test, children were shown a stimulus figure and asked to find it in a series of figures. The results of all the subtests are combined to form three composites: Motor-Reduced Visual Perception, Visual-Motor Integration, and General Visual Perception [23]. DTVP has been validated and proved to be internally consistent when compared to other established tools assessing visual perception, such as Beery-Buktenica Developmental Test of Visual-Motor Integration (VMI) and Test of Visual Perceptual Skills (TVPS-3) [24]. The results yield scores including raw values, age equivalents, percentiles, and composite quotients that provide insight into child's general visual perceptual abilities as well as indicating specific strengths and weaknesses.

### 3.3. MRI Study

Children were subjected to MRI studies using a 1.5T GE HDxt system (General Electric Healthcare, Milwaukee, WI, USA) equipped with an 8-channel head coil. Morphological brain changes were assessed using standard sequences:

(1) Propeller T2 fast spin echo sequence in axial plane (slice thickness 4.0 mm, spacing 2.0 mm, TR 6000 ms, TE 97 ms, FOV 24 cm, and matrix 320 × 320).
(2) T2 FRFSE-XL fast spin echo sequences in sagittal plane (slice thickness 4.0 mm, spacing 2.0 mm, TR 3660 ms, TE 88 ms, FOV 24 cm, and matrix 384 × 224).
(3) T2 FRFSE-XL fast spin echo sequences in coronal plane (slice thickness 4.0 mm, spacing 2.0 mm, TR 4600 ms, TE 88 ms, FOV 24 cm, and matrix 384 × 224).
(4) Propeller T2 FLAIR in axial plane (slice thickness 4.0 mm, spacing 2.0 mm, TR 8000 ms, TE 123 ms, T1 8000 ms, FOV 24 cm, and matrix 288 × 288).
(5) T1 spin echo sequence in axial plane (slice thickness 4.0 mm, spacing 2.0 mm, TR 320 ms, TE 9 ms, FOV 24 cm, and matrix 512 × 224).
(6) GRE T2^*∗*^ gradient echo sequence in axial plane (slice thickness 4.0 mm, spacing 2.0 mm, TR 720 ms, TE 15 ms, flip angle 20, FOV 24 cm, and matrix 320 × 192).
(7) FSPGR T1 gradient echo IR prepared sequence in axial, coronal, and sagittal plane (slice thickness 2.0 mm, spacing −1,0 mm, TR 10 ms, TE 4.4 ms, TI 450 ms, flip angle 12, FOV 20 cm, and matrix 320 × 192).
(8) DWI echo planar imaging (DWEPI) sequence in axial plane (slice thickness 4.0 mm, spacing 2.0 mm, TR 8000 ms, TE 98 ms, FOV 24 cm, and matrix 128 × 128).
(9) DTI echo planar imaging (DWEPI) sequence in axial, coronal, and sagittal plane (slice thickness 5.0 mm, spacing 1.0 mm, TR 8000 ms, TE 109,7 ms, FOV 20 cm, and matrix 128 × 128). The diffusion gradient for *b* = 1000 s/mm^2^ was oriented in 25 directions. For each subject, fractional anisotropy and colored orientation maps were calculated. DTI analysis was performed using Functool image analysis software (GE Healthcare).

All sequences were performed in axial, sagittal, and coronal plane. The diffusion gradient for *b* = 1000 s/mm^2^ was oriented in 25 directions. For each subject, fractional anisotropy and colored orientation maps were calculated. DTI analysis was performed using Functool image analysis software (GE Healthcare).

### 3.4. CC Evaluation

**Figure 1:**
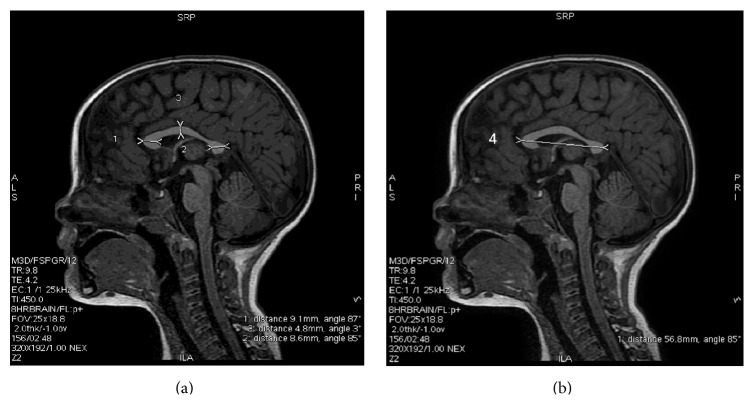
The size of corpus callosum (CC) was determined using the midline sagittal MRI scans. Anterior/genu (1), posterior/tail (2), and medium/tail (3) portions of the CC were measured (a). The CC area was measured manually by drawing a line at the greatest anteroposterior dimension (4) of the CC and another line is drawn perpendicularly at its midpoint. The corpus callosum index (CCI) was calculated as (1 + 2 + 3)/4 (b).

The size of CC was determined using the midline sagittal MRI scans. Anterior/genu, posterior/tail, and medium/tail portions of the CC were measured (Figure 1(a)). The CC area was measured manually by drawing a line at the maximal anteroposterior dimension of the CC and another line is drawn perpendicularly at its midpoint (Figure 1(b)) [25]. The measurements have been performed on a picture storing system on the workstation Advantage Windows (GE Healthcare) (Figures 1(a) and 1(b)). The CC index was calculated as (1 + 2 + 3)/4 following the method presented by Figueira et al. [26].

### 3.5. Analysis of the Microstructure of WM

**Figure 2:**
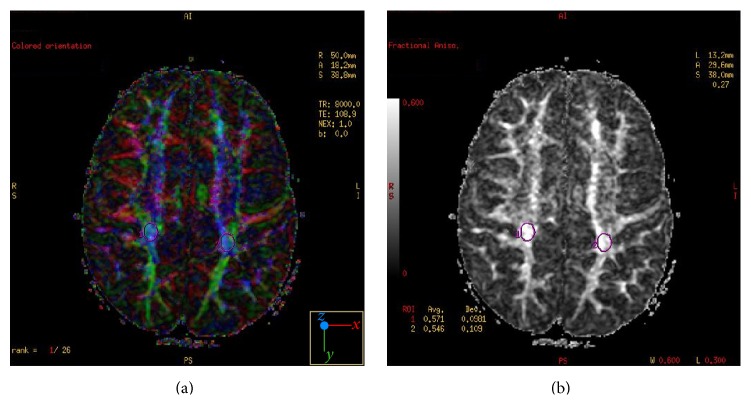
Axial plane images obtained at the level just above the superior margins of the lateral ventricles. The fractional anisotropy (FA) color map (a) and FA grey map (b) were used to determine the regions of interest located in the superior occipital white matter (1, right side, 2, left side).

**Figure 3:**
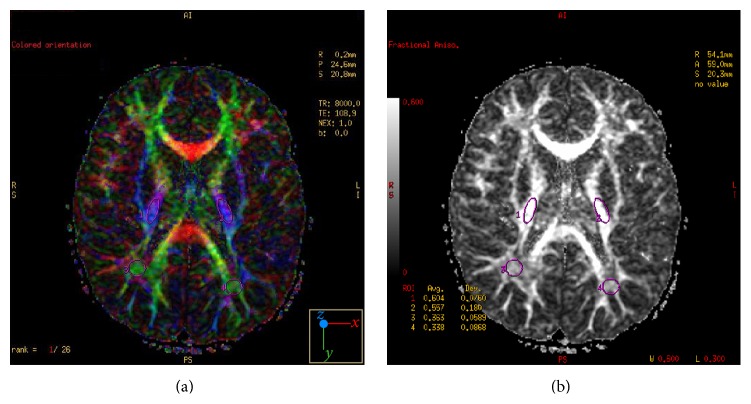
Axial plane images obtained at the level of the basal ganglia and posterior limb of internal capsule. The fractional anisotropy (FA) color map (a) and FA grey map (b) were used to determine the regions of interest located in the middle third of the posterior limbs of internal capsule (1, right side, 2, left side) and occipital white matter (3, right side, 4, left side).

The integrity of WM connections was scrutinized using gradient DTI sequence. To objectively evaluate microstructural changes of WM, six regions of interest were selected: left and right superior occipital white matter (OWM) (Figure 2), left and right inferior OWM, and (as control regions) left and right posterior limbs of the internal capsule (PLIC) (Figure 3). To analyze callosal microstructure/WM integrity three regions of interest were selected, located in genu, body, and splenium area. Apparent diffusion coefficient (ADC), fractional anisotropy (FA), and attenuation coefficient (AC) values were calculated for each region.

The MRI evaluators were not informed about the results of visual examinations.

### 3.6. Outcome Variables

Abnormal stereoscopic vision was the primary outcome variable. Secondary outcome variables included results of the DTVP test.

### 3.7. Statistical Analysis

Student's *t*-test, Mann-Whitney *U* test, or Fisher's exact test was utilized to compare variables between the groups (Table 1). Factors associated with abnormal stereoscopic vision in univariate analyses were entered as covariates for logistic regression analysis. Logistic regression was performed to estimate odds ratios for abnormal stereoscopic vision among former VLBW infants. Data was analyzed using SPSS Software (version 22, 2013, by IBM Corporation, Armonk, NY, USA).

## 4. Results

Sixty-one children with mean birth weight of 1024 g (SD 270 g) were evaluated at the mean age of 48 months (range 42–54). Based on the results of the ophthalmologic evaluation, children were divided into two groups: group A, abnormal stereoscopic vision (*N* = 16), and group B, normal stereoscopic vision (*N* = 45). The comparison of selected demographic variables was shown in Table 1. The groups were similar with respect to age and gender. Children with abnormal stereoscopic vision were significantly more immature and their body mass at birth was lower than in control group. Univariate analysis showed that history of ROP was a significant risk factor for abnormal stereoscopic vision at the age of 4 years in VLBW children.

The comparison of selected CC variables measured on the midline sagittal MRI scans between children with abnormal stereoscopic vision and the control group is shown in Table 2. Children with abnormal stereoscopic vision had significantly smaller length of CC, width of rostrum and main body of CC, and finally smaller estimated CC area. Smaller size of CC was accompanied by lower values of FA.

Table 3 presents results of DWI measurements in the areas of occipital WM and posterior limbs of internal capsule. FA values of inferior right OWM were significantly higher in the control group compared to the group of children with abnormal vision. No other DTI parameters correlated with the stereoscopic vision status. Similar results were obtained using ROP as a covariate in statistical analysis.

**Figure 4:**
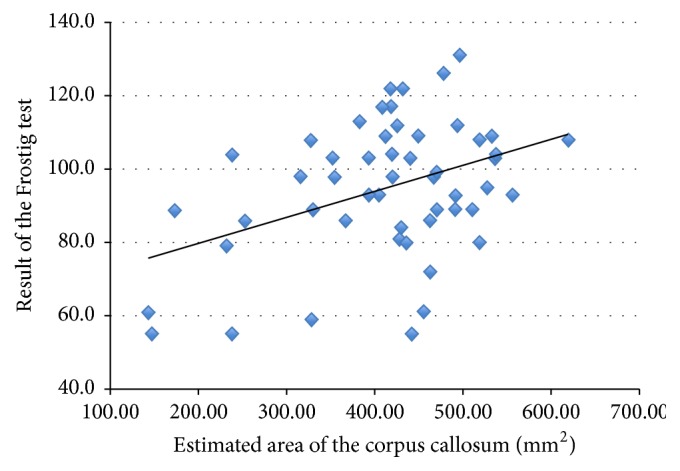
Correlation between estimated corpus callosum area and the result of the Developmental Test of Visual Perception (DTVP-3, Frostig test) score.

**Figure 5:**
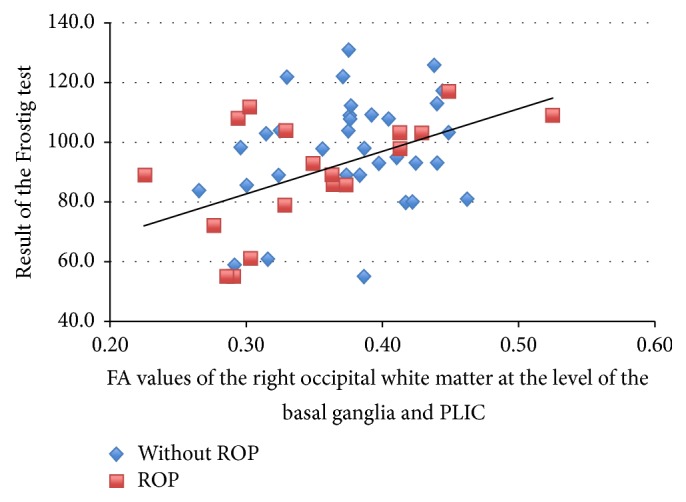
Correlation between fractional anisotropy measurements of the right occipital white matter at the level of basal ganglia and posterior limb of internal capsule and the result of the Developmental Test of Visual Perception (DTVP-3, Frostig test) score in the group of children with and without history of retinopathy of prematurity (ROP, retinopathy of prematurity).

There were significant correlations between the DTVP scores and CC size and FA values of rostrum of CC and FA values in OWM voxels (Table 4).  Figure 4 shows correlation between estimated CC area and the result of the DTVP score. It is important to stress that not only size of CC, but also FA values of anterior and middle part of CC correlated with DTVP scores. The correlation between the DTVP scores and FA measurements did not depend on ROP status (Figure 5).

Finally, a multivariate logistic regression revealed that gestational age and estimated CC area were the only independent risk factors for abnormal stereoscopic vision (Table 5).

## 5. Discussion

This study assessed the relationship between stereoscopic vision, visual perception, and brain microstructure in a cohort of VLBW children at the age of 4 years. The study links abnormalities of stereoscopic vision with the size and structure of CC, stressing its crucial role in visual integration. One of the primary goals of our study was to combine ophthalmologic and psychological assessment with modern imaging techniques in order to develop a prognostic tool for visual perception in VLBW children.

Despite substantial body of data linking macroscopic brain abnormalities with abnormal visual perception it has become clear that not all functional disturbances can be explained by macroscopic data. In this context DTI provides much more detailed information regarding WM microstructure. Immaturity of WM microstructure in preterm neonates has been previously described using DTI [27]. Correlation between optic tract integrity assessed by FA measurement and visual maturity in preterm infants has been reported by Berman et al. [8] and Bassi et al. [28]. Recently Kelly et al. have confirmed a correlation between optic tract microstructure abnormalities and visual impairment in a group of extremely prematurely born adolescents [29]. Low scores of the Developmental Test of Visual-Motor Integration have also been linked with low FA values in the external capsule, posterior part of the internal capsule, and the inferior fasciculus [30].

Our results show that stereoscopic vision may be related to macro- and microstructure of the CC. CC of VLBW children with abnormal stereoscopic vision is more likely to be smaller, compared to controls with normal stereoscopic vision. Most importantly we were able to show that abnormal perceptual performance in VLBW children is associated with microstructure of the CC and microstructure of superior and inferior WM bilaterally. The first convincing data on fundamental role of CC in the interhemispheric communication appeared over fifty years ago [31]. However, until recently there was very little evidence of specific topographic organization of the human CC. Several studies point to the splenium of the CC as the portion containing most occipital callosal connections implicated in visual integration. Recent studies provided proof that the splenium also contains connections from temporal and parietal lobes [32]. Interestingly we were unable to show correlation between splenial FA and stereoscopic vision, but to our knowledge, for the first time, we observed significant relationship between FA values of the rostrum, genu, and body of the CC and stereoscopic vision in VLBW children. We speculate that this finding might suggest a more complex model of interhemispheric visual integration, requiring not only interoccipital but also frontal, parietal, and temporal involvement. However, given a relatively small sample size in our study and the fact that methodology utilized in our protocol is aimed primarily for clinical use, one has to acknowledge that these results are burdened with uncertainty.

The fact that we were able to observe correlation between stereoscopic vision and white matter FA in right but not left occipital WM at the level of the basal ganglia and PLIC may reflect significance of laterality and the key role of the right hemisphere in visual integration, which have been previously reported [2].

Major complications of prematurity such as ROP, PVL, and IVH used to be considered important risk factors for visual perception abnormalities in further life. However, modern development of neonatal care including widespread use of prenatal steroid, less-invasive modalities of respiratory support including CPAP, and routine use of surfactant have significantly reduced prevalence of these complications. Most studies assessing the impact of prematurity on perceptive skills published to date focus on VLBW adolescents [33–35]. Therefore the clinical characteristics of those cohorts and higher prevalence of the abovementioned complications could alter the interpretation of the results. The prevalence of high-grade IVH and PVL in our cohort of VLBW children was low. We were unable to show any relationship between stereoscopic vision and those sequels of prematurity. Similarly, based on logistic regression analysis, ROP was dismissed as a risk factor for abnormal stereoscopic vision. The only two factors that proved to be related to stereoscopic vision impairment were gestational age and structure of the CC. These results point to the importance of more subtle abnormalities as possible factors of long-term cognitive prognosis in survivors of prematurity.

All children participating in our study have been subjected to a thorough and systematic multidisciplinary follow-up that was initiated on discharge and continued throughout the whole observation period. To further objectify our results we introduced blinding in MRI assessment. The person describing the study results was not aware of the patient's data, group assignment, or DTVP and stereoscopic vision tests results.

Our study has some limitations. Lack of control group of healthy term individuals hinders our ability to precisely estimate the impact of the microstructural abnormalities. The tests used for assessment of stereoscopic vision may lack sensitivity in the group of young children. In order to increase the overall chance of detecting abnormalities, three different tests were included in the protocol. Finally, the sample size of the study cohort and the analysis protocol optimized primarily for clinical use preclude drawing large-scale population-wide conclusions. The scope of data accessible for analysis was also limited due to restrictions imposed by the software package that was used.

In conclusion we found a significant correlation between visual perception, stereoscopic vision, and microstructure of the CC in VLBW children at the age of 4. Our results were unrelated to major known complications of prematurity including PVL, IVH, and high-grade ROP. Relationship between FA in rostrum, genu, and body of the CC and visual perception strongly suggests a more complex model of interhemispheric communication and its role in visual integration. Further studies are needed to precisely define a functional map of intercallosal connections in VLBW survivors.

## Supplementary Material

To analyze callosal microstructure/WM integrity three regions of interest were selected, located in genu, body and splenium area. Apparent diffusion coefficient (ADC), fractional anisotropy (FA) and attenuation coefficient (AC) values were calculated for each region. The detailed results of ADC and AC values were presented in Supplementary Table 1.

## Figures and Tables

**Table 1:** Comparison of selected demographic and clinical variables between children with abnormal stereoscopic vision and the control group^a^.

	Abnormal stereoscopic vision (*N* = 16)	Control group (*N* = 45)	*p* value
Birth weight (mean ± SD)	**775** ± **182**	**1079** ± **240**	**<0.01** ^b^
Gestational age (mean ± SD)	**25.9** ± **1.8**	**28.3** ± **2.1**	**<0.01** ^b^
Female	6 (38%)	22 (49%)	0.56^c^
Vaginal delivery	6 (38%)	13 (29%)	0.54^c^
Small for gestational age* *	4 (25%)	7 (16%)	0.46^c^
5 min Apgar score. Median (25th–75th percentile)* *	5 (4–7)	6 (5–7)	0.4^d^
Age at evaluation (months). Median (25th–75th percentile)	48 (45–51)	48 (46–49)	0.9^d^
ROP requiring laser therapy	**12 (75%)**	**10 (22%)**	**<0.01** ^c^
IVH grade III	2 (12.5%)	4 (9%)	0.7^c^
IVH grade IV	0	0	n.a.
PVL	2 (12.5%)	5 (11%)	1.0^c^

^a^Expressed as a number (percentage) of patients unless otherwise indicated; *p* value for Student's *t*-test^b^. Fisher's exact test^c^, Mann-Whitney *U* test^d^.

ROP, retinopathy of prematurity; IVH, intraventricular hemorrhage; PVL, periventricular leukomalacia.

**Table 2:** Comparison of selected corpus callosum variables measured on the midline sagittal MRI scans between children with abnormal stereoscopic vision and the control group^a^.

	Abnormal stereoscopic vision (*N* = 16)	Control group (*N* = 45)	Student's *t*-test *p* value	*p* value adjusted for confounding variables^b^
Rostrum/genu (mm)	**6.96 (1.9)**	**8.7 (1.6)**	**0.01**	**0.018**
Body (mm)	**5.97 (2.07)**	**8.3 (1.6)**	**<**0.001	**0.003**
Splenium (mm)	4.53 (1.5)	4.98 (1.1)	0.2	0.89
CC length (mm)	**53 (6.2)**	**61 (4.1)**	**<0.001**	**<0.001**
Estimated CC area (mm^2^)	**314 (106)**	**446 (79)**	**<0.001**	**0.001**
FA value of rostrum/genu	**0.7 (0.09)**	**0.79 (0.07)**	**<0.001**	**0.037**
FA value of CC body	**0.74 (0.13)**	**0.82 (0.09)**	**0.03**	0.46
FA value of CC splenium	0.67 (0.13)	0.71 (0.11)	0.2	0.59

^a^Expressed as mean (SD).

^b^Analysis adjusted for birth weight and gestational age.

FA, fractional anisotropy; CC, corpus callosum.

**Table 3:** Comparison of selected fractional anisotropy (FA) values measured on the axial MRI scans between children with abnormal stereoscopic vision and the control group^a^.

	Abnormal stereoscopic vision (*N* = 16)	Control group (*N* = 45)	Student's *t*-test *p* value
FA values of the left OWM at the level just above the superior margins of the lateral ventricles	0.38 (0.07)	0.39 (0.05)	0.6

FA values of the right OWM at the level just above the superior margins of the lateral ventricles	0.38 (0.07)	0.38 (0.06)	0.9

FA values of the left OWM at the level of the basal ganglia and PLIC	0.34 (0.06)	0.37 (0.06)	0.1

FA values of the right OWM at the level of the basal ganglia and PLIC	**0.30 (0.06)**	**0.36 (0.06)**	**0.01**

FA values of the left PLIC at the level of the basal ganglia and PLIC	0.55 (0.04)	0.55 (0.05)	0.9

FA values of the right PLIC at the level of the basal ganglia and PLIC	0.54 (0.05)	0.55 (0.06)	0.9

FA, fractional anisotropy; PLIC, posterior limb of internal capsule; OWM, occipital white matter; ^a^Expressed as a mean (SD).

**Table 4:** Correlation between the results of DTVP-3 and selected MRI variables in the group of 4-year-old very low birth weight infants.

	*R*	*p* value
Rostrum/genu of CC	**0.45**	**0.001**
Body of CC	**0.31**	**0.02**
Splenium of CC	0.18	0.2
CC length	**0.32**	**0.02**
Estimated CC area	**0.4**	**0.003**
FA value of rostrum/genu	**0.38**	**0.01**
FA value of CC body	**0.34**	**0.02**
FA value of CC splenium	0.18	0.2
FA value of the left superior WM	**0.38**	**0.01**
FA value of the right superior WM	**0.29**	**0.05**
FA value of the left inferior WM	**0.39**	**0.01**
FA value of the right inferior WM	**0.42**	**0.003**
FA value of the left PLIC	0.16	0.3
FA value of the right PLIC	−0.1	0.4

CC, corpus callosum, FA, fractional anisotropy; WM, white matter, PLIC, posterior limb of internal capsule.

**Table 5:** Logistic regression analysis with abnormal stereoscopic vision as the outcome variable.

Factor	OR (95% CI)	*p* value
Gestational age (per week)	0.59 (0.36–0.98)	0.042
ROP	0.91 (0.3–2.73)	0.87
Estimated CC area (per 100 mm^2^)	0.31 (0.13–0.74)	0.009

ROP, retinopathy of prematurity; OR, odds ratio; CI, confidence interval; CC, corpus callosum.

## References

[1] Geldof C. J. A., Oosterlaan J., Vuijk P. J., de Vries M. J., Kok J. H., van Wassenaer-Leemhuis A. G. (2014). Visual sensory and perceptive functioning in 5-year-old very preterm/very-low-birthweight children. *Developmental Medicine and Child Neurology*.

[2] O'Connor A. R., Stephenson T. J., Johnson A. (2004). Visual function in low birthweight children. *British Journal of Ophthalmology*.

[3] Dutton G. N., Jacobson L. K. (2001). Cerebral visual impairment in children. *Seminars in Neonatology*.

[4] Stiers P., van den Hout B. M., Haers M. (2001). The variety of visual perceptual impairments in pre-school children with perinatal brain damage. *Brain and Development*.

[5] Van den Hout B. M., Stiers P., Haers M. (2000). Relation between visual perceptual impairment and neonatal ultrasound diagnosis of haemorrhagic-ischaemic brain lesions in 5-year-old children. *Developmental Medicine and Child Neurology*.

[6] Eikenes L., Løhaugen G. C., Brubakk A.-M., Skranes J., Håberg A. K. (2011). Young adults born preterm with very low birth weight demonstrate widespread white matter alterations on brain DTI. *NeuroImage*.

[7] Martinussen M., Flanders D. W., Fischl B. (2009). Segmental brain volumes and cognitive and perceptual correlates in 15-year-old adolescents with low birth weight. *Journal of Pediatrics*.

[8] Berman J. I., Glass H. C., Miller S. P. (2009). Quantitative fiber tracking analysis of the optic radiation correlated with visual performance in premature newborns. *American Journal of Neuroradiology*.

[9] Geldof C. J. A., van Wassenaer A. G., de Kieviet J. F., Kok J. H., Oosterlaan J. (2012). Visual perception and visual-motor integration in very preterm and/or very low birth weight children: a meta-analysis. *Research in Developmental Disabilities*.

[10] Rose S. A., Feldman J. F., Jankowski J. J., Van Rossem R. (2011). Basic information processing abilities at 11 years account for deficits in IQ associated with preterm birth. *Intelligence*.

[11] Krägeloh-Mann I., Toft P., Lunding J., Andresen J., Pryds O., Lou H. C. (1999). Brain lesions in preterms: origin, consequences and compensation. *Acta Paediatrica*.

[12] Van den Hout B. M., de Vries L. S., Meiners L. C. (2004). Visual perceptual impairment in children at 5 years of age with perinatal haemorrhagic or ischaemic brain damage in relation to cerebral magnetic resonance imaging. *Brain & Development*.

[13] Soares J. M., Marques P., Alves V., Sousa N. (2013). A hitchhiker's guide to diffusion tensor imaging. *Frontiers in Neuroscience*.

[14] Pandit A. S., Ball G., Edwards A. D., Counsell S. J. (2013). Diffusion magnetic resonance imaging in preterm brain injury. *Neuroradiology*.

[15] Dougherty R. F., Koch V. M., Brewer A. A., Fischer B., Modersitzki J., Wandell B. A. (2003). Visual field representations and locations of visual areas v1/2/3 in human visual cortex. *Journal of Vision*.

[16] Dougherty R. F., Ben-Shachar M., Bammer R., Brewer A. A., Wandell B. A. (2005). Functional organization of human occipital-callosal fiber tracts. *Proceedings of the National Academy of Sciences of the United States of America*.

[17] Saenz M., Fine I. (2010). Topographic organization of V1 projections through the corpus callosum in humans. *NeuroImage*.

[18] Rockland K. S., Pandya D. N. (1986). Topography of occipital lobe commissural connections in the rhesus monkey. *Brain Research*.

[19] Hubel D. H., Wiesel T. N. (1967). Cortical and callosal connections concerned with the vertical meridian of visual fields in the cat. *Journal of Neurophysiology*.

[20] Binder J. R., Mohr J. P. (1992). The topography of callosal reading pathways. A case-control analysis. *Brain*.

[21] Todorow M., DeSouza J. F., Banwell B. L., Till C. (2014). Interhemispheric cooperation in global-local visual processing in pediatric multiple sclerosis. *Journal of Clinical and Experimental Neuropsychology*.

[22] Ancona C., Stoppani M., Odazio V., La Spina C., Corradetti G., Bandello F. (2014). Stereo tests as a screening tool for strabismus: which is the best choice?. *Clinical Ophthalmology*.

[23] Frostig M., Lefever D. W., Whittlesey J. R. (1961). A developmental test of visual perception for evaluating normal and neurologicallyhandicapped children. *Perceptual and Motor Skills*.

[24] Brown T., Hockey S. C. (2013). The validity and reliability of developmental test of visual perception-2nd edition (DTVP-2). *Physical & Occupational Therapy in Pediatrics*.

[25] Kosar M. I., Erdil F. H., Sabanciogullari V., Karacan K., Cimen M., Atalar M. (2012). Morphometry of corpus callosum related with gender and age: magnetic resonance imaging study. *Pakistan Journal of Medical Sciences*.

[26] Figueira F. F. A., Dos Santos V. S., Figueira G. M. A., Da Silva Â. C. M. (2007). Corpus callosum index: a practical method for long-term follow-up in multiple sclerosis. *Arquivos de Neuro-Psiquiatria*.

[27] Anjari M., Srinivasan L., Allsop J. M. (2007). Diffusion tensor imaging with tract-based spatial statistics reveals local white matter abnormalities in preterm infants. *NeuroImage*.

[28] Bassi L., Ricci D., Volzone A. (2008). Probabilistic diffusion tractography of the optic radiations and visual function in preterm infants at term equivalent age. *Brain*.

[29] Kelly C. E., Cheong J. L. Y., Molloy C. (2014). Neural correlates of impaired vision in adolescents born extremely preterm and/or extremely low birthweight. *PLoS ONE*.

[30] Skranes J., Vangberg T. R., Kulseng S. (2007). Clinical findings and white matter abnormalities seen on diffusion tensor imaging in adolescents with very low birth weight. *Brain*.

[31] Myers R. E. (1956). Function of corpus callosum in interocular transfer. *Brain*.

[32] Park H.-J., Kim J. J., Lee S. K. (2008). Corpus callosal connection mapping using cortical gray matter parcellation and DT-MRI. *Human Brain Mapping*.

[33] Sripada K., Løhaugen G. C., Eikenes L. (2015). Visual-motor deficits relate to altered gray and white matter in young adults born preterm with very low birth weight. *NeuroImage*.

[34] Molloy C. S., Wilson-Ching M., Anderson V. A., Roberts G., Anderson P. J., Doyle L. W. (2013). Visual processing in adolescents born extremely low birth weight and/or extremely preterm. *Pediatrics*.

[35] Lindqvist S., Skranes J., Eikenes L. (2011). Visual function and white matter microstructure in very-low-birth-weight (VLBW) adolescents—a DTI study. *Vision Research*.

