# Supplementary material for: Relationship between Stereoscopic Vision, Visual Perception, and Microstructure Changes of Corpus Callosum and Occipital White Matter in the 4-Year-Old Very Low Birth Weight Children
Source: Biomed Res Int. 2015 Sep 16;2015:842143. doi: 10.1155/2015/842143 (PMC4588345; doi:10.1155/2015/842143)
Supplement: Supplementary file 1 — To analyze callosal microstructure/WM integrity three regions of interest were selected, located in genu, body and splenium area. Apparent diffusion coefficient (ADC), fractional anisotropy (FA) and attenuation coefficient (AC) values were calculated for each region. The detailed results of ADC and AC values were presented in Supplementary Table 1. [file 842143.f1.docx]

Suppl. Table 1. Comparison of selected DTI parameters between children with abnormal stereoscopic vision and the control group a

|  | Abnormal stereoscopic vision  (N=16) | Control group  (N=45) | Student’s t test p value |
| --- | --- | --- | --- |
| CC rostrum/genu | | | |
| ADC (x10^-3^ mm^2^/s) | 0.94 (0.16) | 0.86 (0.09) | 0.053 |
| AC (s/mm^2^) | 0.27 (0.07) | 0.30 (0.04) | 0.14 |
| CC body | | | |
| ADC (x10^-3^ mm^2^/s) | 0.90 (0.17) | 0.80 (0.11) | 0.057 |
| AC (s/mm^2^) | 0.29 (0.06) | 0.32 (0.05) | 0.083 |
| CC splenium | | | |
| ADC (x10^-3^ mm^2^/s) | 0.98 (0.24) | 0.85 (0.09) | 0.06 |
| AC (s/mm^2^) | 0.28 (0.07) | 0.31 (0.07) | 0.072 |
| Left OWM at the level just above the superior margins of the lateral ventricles | | | |
| ADC (x10^-3^ mm^2^/s) | 0.81 (0.04) | 0.81 (0.04) | 0.48 |
| AC (s/mm^2^) | 0.30 (0.05) | 0.30 (0.04) | 0.84 |
| Right OWM at the level just above the superior margins of the lateral ventricles | | | |
| ADC (x10^-3^ mm^2^/s) | 0.81 (0.05) | 0.80 (0.07) | 0.67 |
| AC (s/mm^2^) | 0.31 (0.04) | 0.31 (0.04) | 0.93 |
| Left OWM at the level of the basal ganglia and PLIC | | | |
| ADC (x10^-3^ mm^2^/s) | 0.90 (0.09) | 0.86 (0.05) | 0.06 |
| AC (s/mm^2^) | 0.27 (0.04) | 0.28 (0.06) | 0.37 |
| Right OWM at the level of the basal ganglia and PLIC | | | |
| ADC (x10^-3^ mm^2^/s) | 0.90 (0.09) | 0.86 (0.06) | 0.08 |
| AC (s/mm^2^) | 0.27 (0.04) | 0.29 (0.06) | 0.15 |
| Left PLIC at the level of the basal ganglia and PLIC | | | |
| ADC (x10^-3^ mm^2^/s) | 0.77 (0.03) | 0.74 (0.10) | 0.23 |
| AC (s/mm^2^) | 0.33 (0.03) | 0.34 (0.03) | 0.34 |
| Right PLIC at the level of the basal ganglia and PLIC | | | |
| ADC (x10^-3^ mm^2^/s) | 0.76 (0.03) | 0.76 (0.03) | 0.78 |
| AC (s/mm^2^) | 0.33 (0.03) | 0.32 (0.05) | 0.65 |

^a^ Expressed as a mean (SD)

ADC – apparent diffusion coefficient;

AC – attenuation coefficient

PLIC – posteriori limb of internal capsule
